# Supplementary material for: Synthesis of Novel Nilotinib Analogues and Biological Evaluation of Their Antiplatelet Activity and Functionality towards Cancer Cell Proliferation In Vitro
Source: Pharmaceuticals (Basel). 2024 Mar 7;17(3):349. doi: 10.3390/ph17030349 (PMC10974042; doi:10.3390/ph17030349)
Supplement: Supplementary file 1 [file pharmaceuticals-17-00349-s001.zip › pharmaceuticals-2866024-supplementary.pdf]

Supporting Information

Synthesis of Novel Nilotinib Analogues and Biological Evaluation of Their Antiplatelet Activity and Functionality towards Cancer Cell Proliferation, In Vitro

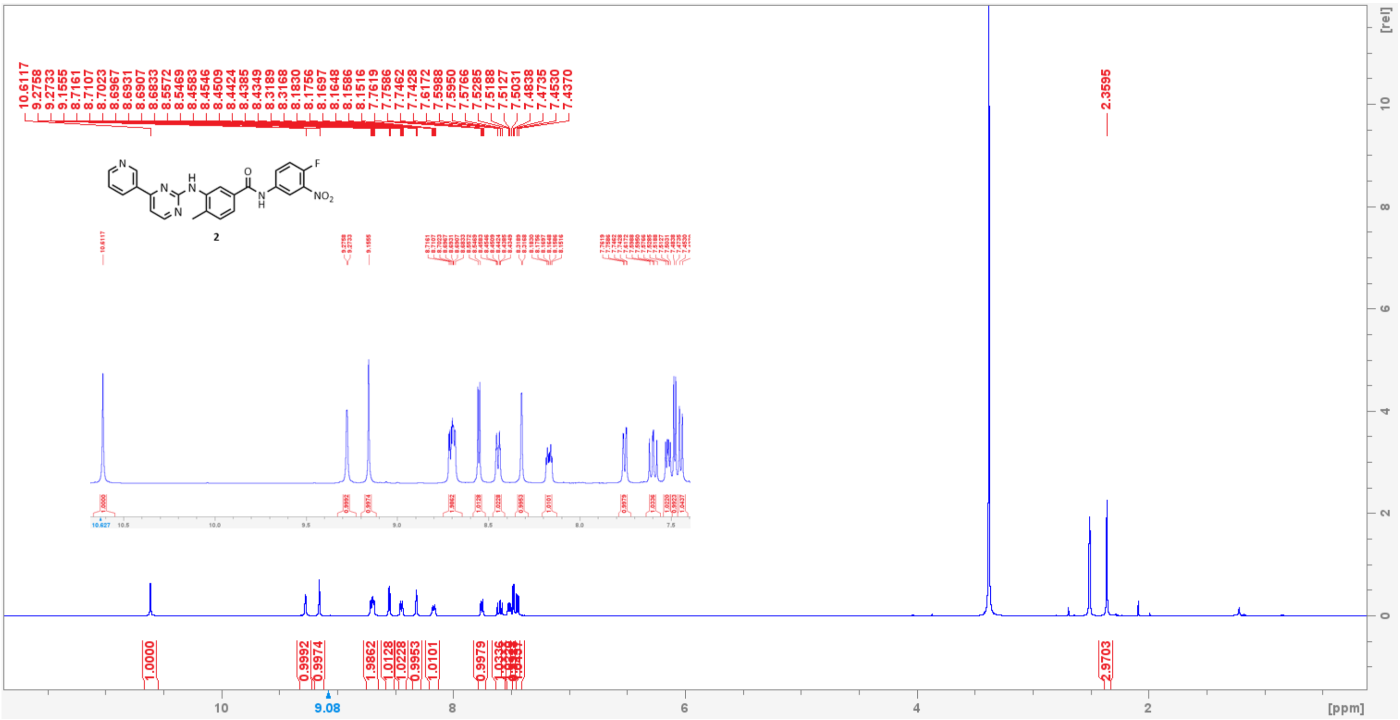

Figure. S1. ^{1}H NMR (500 MHz, DMSO-d_6) of the final compound-imatinib analogue **2**.

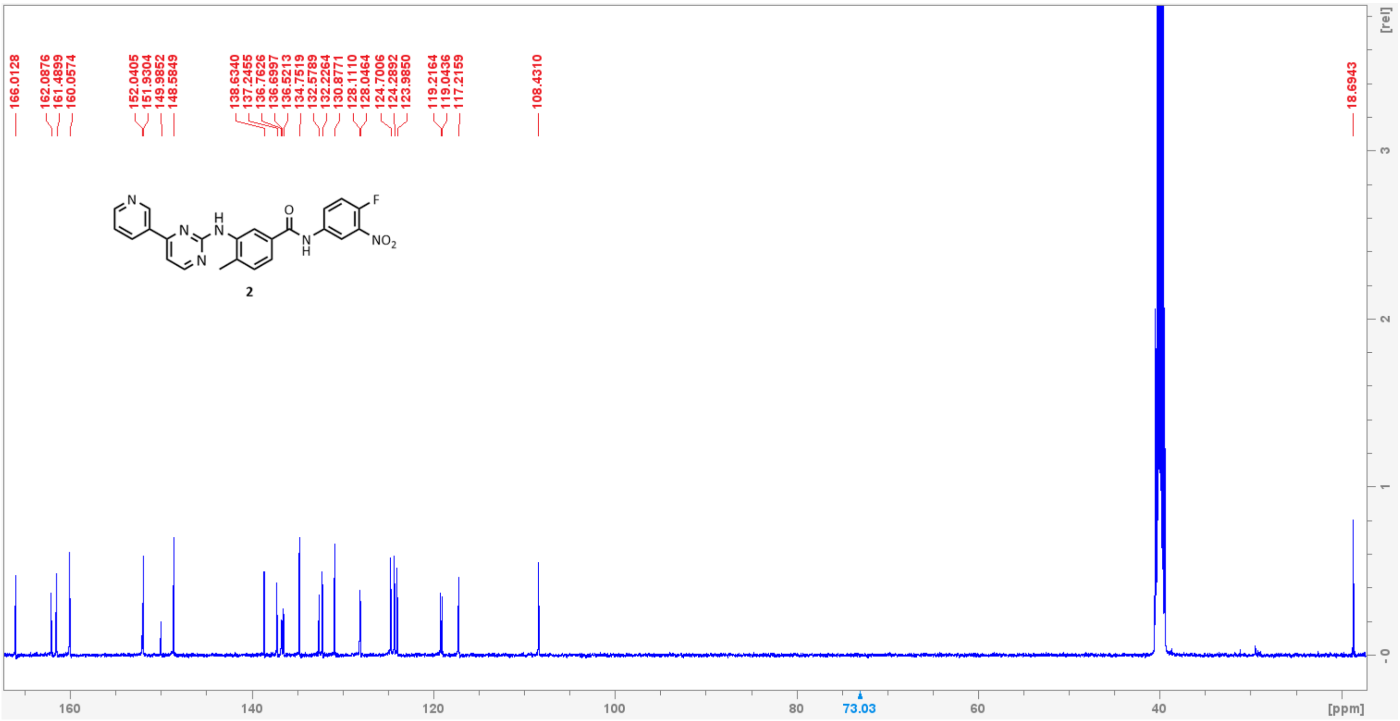

Figure S2. ^{13}C NMR (125 MHz, DMSO-d_6) of the final compound-imatinib analogue **2**.

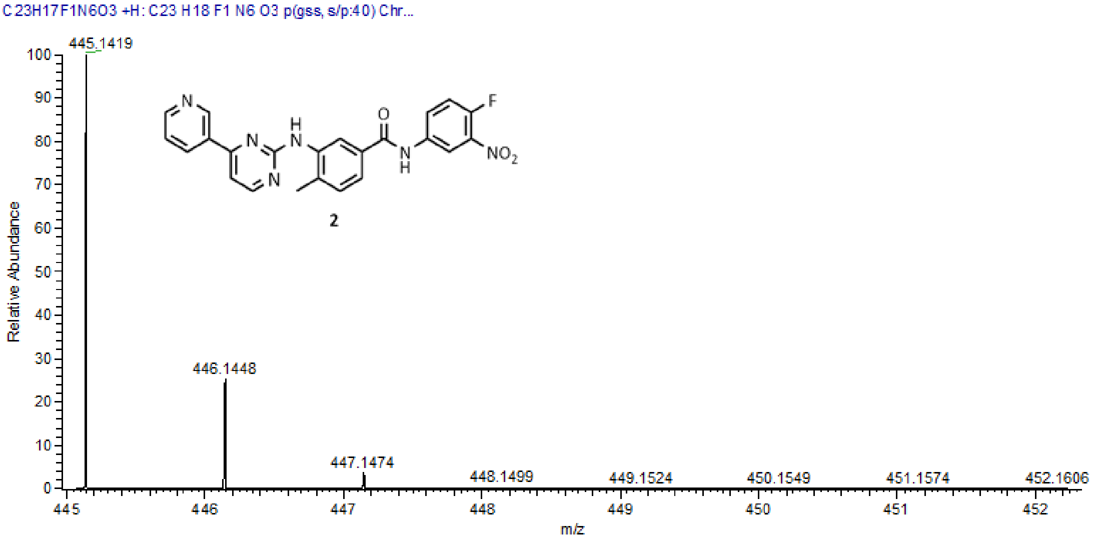

Figure S3. High resolution ESI-MS of the final compound-imatinib analogue **2**.

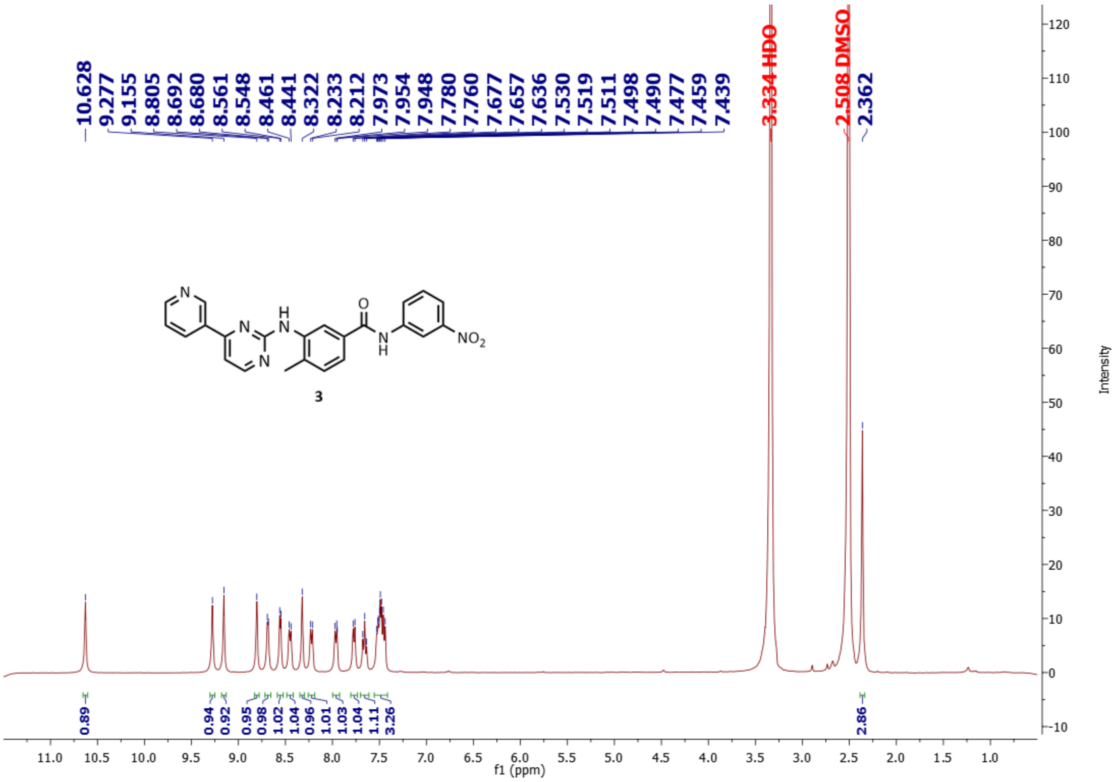

Figure S4. ^{1}H NMR (400 MHz, DMSO-d_6) of the final compound-imatinib analogue **3**.

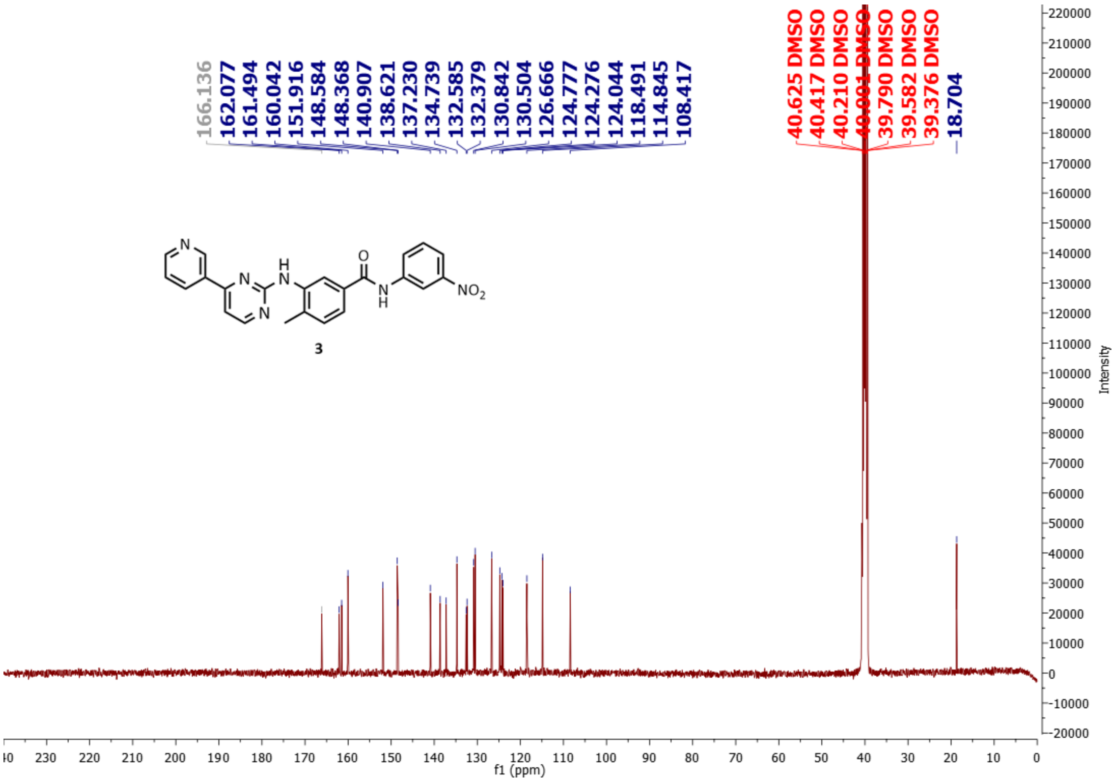

Figure S5. ^{13}C NMR (100 MHz, DMSO-d_6) of the final compound-imatinib analogue **3**.

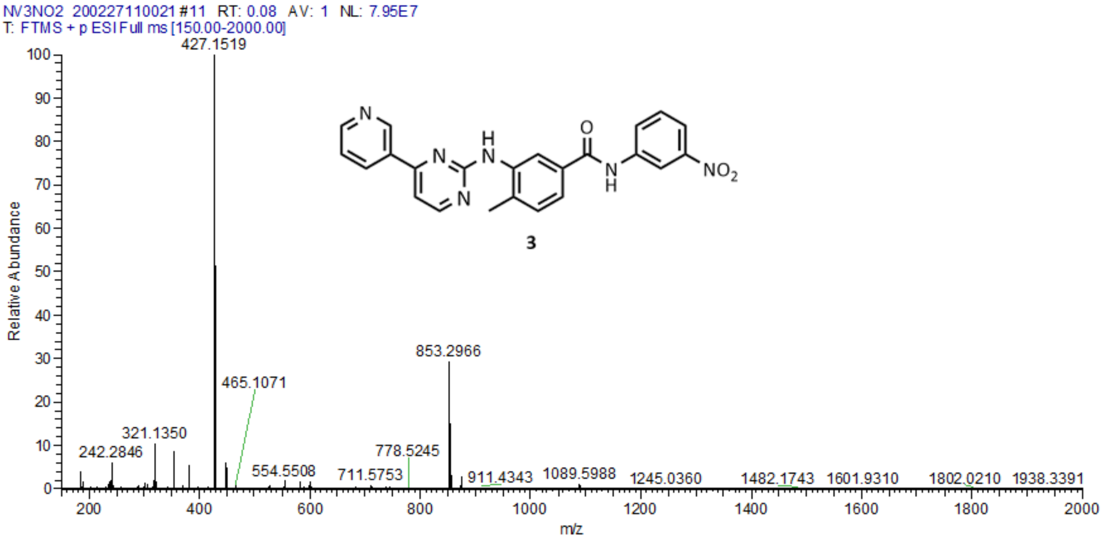

Figure S6. High resolution ESI-MS of the final compound-imatinib analogue **3**.
